# A Dietary Mixture of Oxysterols Induces In Vitro Intestinal Inflammation through TLR2/4 Activation: The Protective Effect of Cocoa Bean Shells

**DOI:** 10.3390/antiox8060151

**Published:** 2019-05-31

**Authors:** Daniela Rossin, Letricia Barbosa-Pereira, Noemi Iaia, Gabriella Testa, Barbara Sottero, Giuseppe Poli, Giuseppe Zeppa, Fiorella Biasi

**Affiliations:** 1 Department of Clinical and Biological Sciences, University of Turin, 10043 Orbassano (Turin), Italy; d.rossin@unito.it (D.R.); noemi.iaia@unito.it (N.I.); gabriella.testa@unito.it (G.T.); barbara.sottero@unito.it (B.S.); giuseppe.poli@unito.it (G.P.); 2 Department of Agricultural, Forestry, and Food Sciences (DISAFA), 10095 Grugliasco (Turin), Italy; letricia.barbosapereira@unito.it (L.B.-P.); giuseppe.zeppa@unito.it (G.Z.)

**Keywords:** dietary oxysterols, Toll-like receptors, IL-8, IFNβ, TNFα, phenolic extract, cocoa bean shell, Honduras cocoa, (−)-epicatechin, tannins

## Abstract

Background: Exaggerated Toll-like receptor (TLR)-mediated immune and inflammatory responses play a role in inflammatory bowel diseases. This report deals with the ability of a mixture of oxysterols widely present in cholesterol-rich foods to induce in vitro intestinal inflammation through TLR up-regulation. The anti-inflammatory action of four cocoa bean shell (CBS) extracts with different polyphenol content, was tested. Methods: Differentiated intestinal CaCo-2 cells were treated with a dietary oxysterol mixture (Oxy-mix) (60 µM). The expression and activation of TLR2 and TLR4, as well as the production of their downstream signaling effectors IL-8, IFNβ and TNFα were analyzed in the presence or absence of TLR antibodies. Honduras CBS extracts were characterized for their polyphenol contents; their anti-inflammatory action was analyzed in CaCo-2 cells treated with Oxy-mix. Results: Oxysterol-dependent TLR-2 and TLR4 over-expression and activation together with cytokine induction were abolished by blocking TLRs with specific antibodies. Polyphenol-rich CBS extracts consisting of high quantities of (−)-epicatechin and tannins also prevented TLR induction. Conclusions: TLR2 and TLR4 mainly contribute to inducing oxysterol-dependent intestinal inflammation. The fractionation method of CBS allowed the recovery of fractions rich in (−)-epicatechin and tannins able to counteract oxysterol-induced inflammation, thus highlighting the beneficial biological potential of specific CBS extracts.

## 1. Introduction

Toll-like receptors (TLRs) are the main pattern recognition receptors, which mediate innate immunity and inflammatory responses against pathogen-associated molecular patterns (PAMPs) and damage-associated molecular patterns (DAMPs). The altered activation of these receptors plays a role in the pathogenesis of inflammatory intestinal diseases such as inflammatory bowel diseases (IBDs) [1]. At a cellular level, inflammatory processes triggered by defective or exaggerated TLR-mediated response to luminal antigens lead to severe intestinal layer damage. Therefore, altered crosstalk between intestinal epithelial cells and local immune cells is likely to cause intestinal homeostasis breakdown.

Changes in eating habits in developed countries, which have adopted the so-called Western diet, rich in fats and poor in vegetables, are considered a risk factor for human intestinal pathologies, as they are able to influence the degree of inflammation. For instance, a more severe disease course in the adult and young IBD community has been positively associated with increased obesity [2]. Nutritional strategies that reduce fat intake in order to ameliorate IBD patient outcomes have been proposed [3,4]. Most of these interventions have provided evidence of the influence of diet on the control of TLR-dependent host immune response against bacterial and dietary antigens [5].

Dietary fats mainly consist of animal-derived fats, including cholesterol and cholesterol oxidation products named oxysterols. Food-derived oxysterols are characterized by spontaneous oxygenation in the cholesterol ring structure; 7α- and 7β-hydroxycholesterol, 7-ketocholesterol and α/β-epoxycholesterols represent the main oxysterols produced by cholesterol auto-oxidation, and they are always present in food (for a more comprehensive chemical structure of the main diet-derived oxysterols see Figure S1) [6].

These oxysterols are reported to be most common in cholesterol-rich food as a mixture [7] in concentrations ranging from 10 to 100 μM [8,9].

In our previous studies, we found that increasing concentrations of a mixture containing the main dietary oxysterols were able to induce correspondingly increasing pro-inflammatory activities—60 µM was found to be the highest concentration of mixture able to induce strong pro-inflammatory action and cell layer permeability alteration without exerting cytotoxic effect [10,11,12].

The cocoa bean shell (CBS) is the main by-product generated after the roasting and husking of cocoa beans, and it represents a serious disposal issue in the cocoa sector. CBS is already on the market as an ingredient for herbal infusion bags, such as the “ChocoTea” infusion bags from Valberbe^®^ or the “Choco” tisane bags from YogiTea^®^. Moreover, recent studies have recognized that CBS might also be a source of fibers, minerals, polyphenols, and methylxanthines with potential health benefits that can be used as an ingredient for functional food and beverages [13,14,15].

An increasing number of studies have claimed a connection between different eating habits and quantitative/qualitative changes in intestinal microbiota [16,17]. Dietary lipids may influence changes in inflammatory microbial factors, thus leading to the up-regulation of TLR pathways [18,19]. Although TLR ligands are considered of bacterial origin, they may also come from diet or the host [20].

Therefore, intestinal TLRs that recognize lipids and lipopeptides, such as TLR2 (mainly recognizing lipoproteins expressed on the outer membrane of gram-positive bacteria) and TLR4 (recognizing Gram-negative bacteria lipopolysaccharide) [21,22] could also have affinity for other ligands of lipid origin such as oxysterols.

In this work, we investigated the in vitro ability of a dietary oxysterol mixture representative of a hyper-cholesterol diet to induce TLR2 and/or TLR4 expression. We also analyzed if the activation of these receptors is required in mediating oxysterol inflammatory response on differentiated CaCo-2 cells, an experimental model that well mimics the intestinal mucosa monolayer.

Furthermore, based on the evidence of the beneficial effects of phenolic compounds in counteracting the inflammatory response in intestinal cells [23], we tested their action on modulating oxysterol-dependent-TLR expression/activation. In particular, we focused on the activity of cocoa bean shells (CBS) [24], a widely produced and potentially recyclable food industry by-product that is rich in phenolic compounds.

## 2. Materials and Methods

### 2.1. Chemicals

Unless otherwise specified, reagents and chemicals were obtained from Sigma Aldrich (Milan, Italy). Dulbecco's modified Eagle's medium (DMEM) with high glucose content and fetal bovine serum (FBS), were from Euroclone S.p.A (Milan, Italy), and TRIzol reagent was from Invitrogen (San Giuliano Milanese, Italy). High-Capacity Complementary DNA (Cdna) reverse transcription kit, TaqMan gene expression assay kits for human TLR2, TLR4 and β-actin, TaqMan Fast Universal PCR (polymerase chain reaction) master mix, and TaqMan Array 96-well plates were obtained from Applied Biosystems (Monza, Italy). Six-well plates were from VWR International s.r.l. (Milan, Italy). Oxysterols (5α,6α-epoxycholesterol (α-epox), 5β,6β-epoxycholesterol (β-epox), 7-ketocholesterol (7K), 7α-hydroxycholesterol (7α-HC), 7β-hydroxycholesterol (7β-HC)) were from Avanti Polar Lipids (Alabaster, AL, USA). Bio-Rad Protein Assay Dye reagent for protein evaluation was from Bio-Rad (Milan, Italy).

The Human Tumor necrosis factor-α (TNF-α) ELISA (Enzyme-Linked Immunosorbent Assay) kit was obtained from PeProtech (DBA Italia s.r.l., Milan, Italy), the DuoSet ELISA kit for human IL-8 detection was from R&D System, Space Import Export (Milan, Italy) and the Human Interferon –β (IFN-β) ELISA kit was from Pbl (Tebu-Bio Italia s.r.l., Milan, Italy).

Ethanol (≥99.8%), sodium hydroxide (1 M), protocatechuic acid (>97%) (−)-epicatechin (> 90%), gallic acid (≥98.0%) and procyanidin B2 (≥98.5%) (PCB2) were obtained from Fluka (Milan, Italy). Ultrapure water was prepared in a Milli-Q filter system (Millipore, Milan, Italy).

### 2.2. Differentiated CaCo-2 Cell Culture and Treatment

Human colorectal adenocarcinoma CaCo-2 cells were obtained from the Cell Bank Interlab Cell Line Collection (Genoa, Italy). Six-well plates were used for cell culture. The cells (1 × 10^6^/mL density) were grown in DMEM supplemented with 10% heat-inactivated FBS, 1% antibiotic/antimycotic solution (100 U/mL penicillin, 0.1 mg/mL streptomycin, 250 ng/mL amphotericin B and 0.04 mg/mL gentamicin) at 37 °C and 5% CO_2_ humidified atmosphere. CaCo-2 cells (passages 15–20) were cultured until reaching confluence (about 5 days), and were then grown for further 18 days to allow spontaneous differentiation. Cell medium was replaced thrice a week during the culture time [25]. After overnight serum starvation, the cell medium was replaced with DMEM containing 5% FBS, and differentiated CaCo-2 cells were treated with oxysterol mixture (Oxy-mix) (60 μM final concentration) at 37 °C for different hours depending on the analyses. The percentage composition of the Oxy-mix used (42.96% 7K, 32.3% α-epox, 5.76% β-epox, 4.26% 7α-HC and 14.71% 7β-HC) was calculated on Plat and coworkers’ observation. The authors showed that by heating cholesterol for 3 h at 180 °C, about 95% of oxysterols were produced. This percentage of the oxysterols produced contained about 40% 7-ketocholesterol, 35% of two epoxides, and 25% of two hydroxides [7]. The concentration of the Oxy-mix was calculated using 403 g/mol average as molecular weight, the molarity of each component was: 25.8 μM 7K, 19.4 μM α-epox, 3.4 μM β-epox, 2.6 μM 7α-HC, 8.8 μM 7β-HC. In some experiments, cells were pre-treated for 1 h with anti-TLR2 antibody (0.2 μg/mL) and anti-TLR4 antibody (0.2 μg/mL).

### 2.3. Extraction and Fractionation of CBS Compounds

CBS samples produced from Honduras cocoa beans were kindly supplied by Guido Castagna S.r.l. (Turin, Italy). The CBS were ground in an ultra-centrifugal mill Retsch ZM 200 (Retsch Gmbh, Haan, Germany) in order to obtain a uniform powder with a particle size of 250 µm.

**Scheme 1:**
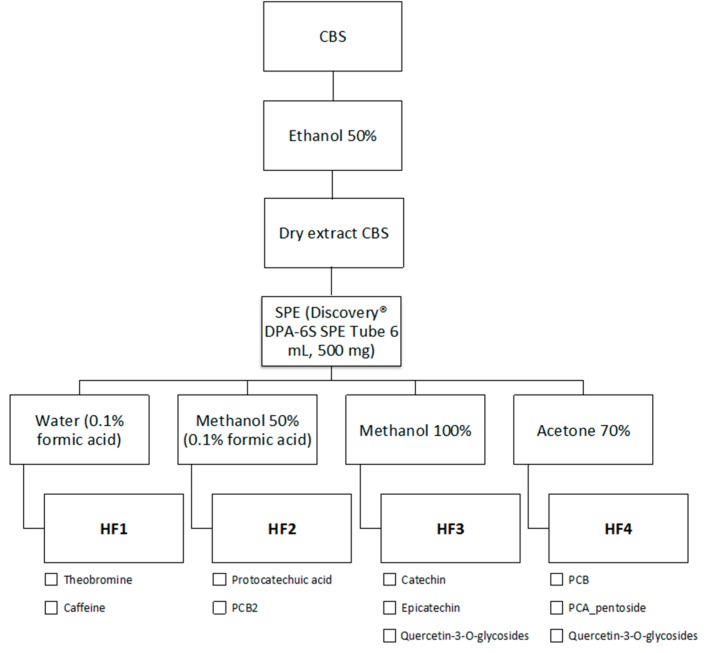
Schematic representation of the extraction and purification procedures to yield the cocoa bean shell (CBS) bioactive compounds. HF1–4: Honduras-Fraction 1–4; PCB: procyanidin B type; PCB2: procyanidin B2; PCA_pentoside: Procyanidin A-type Pentoside.

Fifty g of CBS powder was extracted with 1 l ethanol-water mixture (50:50 *v*/*v*), according to Barbosa-Pereira [15]. Ethanol/water was selected as it is the most suitable solvent for biological application in food ingredients/additives. In addition, the methodology was developed taking into account the implementation of the recovery process at industrial scale. Extraction was performed at 25 °C under constant agitation using Ultra-turrax^®^ stirrer for 1 h. The extract was filtered under vacuum using Whatman^®^ No. 4 qualitative filter paper. Ethanol was evaporated under vacuum at 40 °C in a rotary evaporator, and then the water was eliminated by lyophilization. Finally, the dry extract was redissolved in water in a concentration of 50 mg/mL for a further fractionation/purification step. CBS compound fractionation and purification were performed by using Discovery DPA-6S cartridges (Supelco, USA) containing 500 mg of polyamide sorbent in 6 mL tubes, fitted with frits, in SPE (solid-phase extraction) vacuum manifold (Phenomenex, Castel Maggiore, Italy). These SPE cartridges were specifically to separate phenolics (small molecules) from tannins (higher molecules). However, using different solvents/mix of solvents within intermediate steps, it is possible to separate different groups of compounds. Cartridges were activated with 5 mL methanol, and then preconditioned with 5 mL Mili-Q water. Afterwards, 500 µl of crude extract was loaded on the cartridge and fractionated using 5 mL different solvents or solvent mix as follows: 100% water containing 0.1% formic acid to elute methylxanthines (caffeine and theobromine) (Honduras-Fraction 1, HF1); intermediate polarity methanol-water (50:50 *v*/*v*) (HF2); 100% methanol (HF3) to separate different type of phenolic compounds; and finally acetone-water (70:30 *v*/*v*) to elute all tannins that were retained in the column to clean the column and yield the fourth fraction (HF4) (Scheme 1). The fractionation experiments were performed 24 times and all fractions were collected together according to the type of solvent used in order to have enough quantity of each fraction for chemical and cell experiments (12 each). The organic solvents were dried under nitrogen at 35 °C and constant rotatory agitation at 300 rpm (evaporator/concentrator system Glas-Col^®^ LLC, Terre Haute, USA). Then, an additional amount of ultrapure water was added to the fractions to eliminate the residues of organic solvent during lyophilisation.

For the chemical analysis, each fraction was reconstituted with methanol-water (50:50 *v*/*v*) containing 0.1% formic acid. The fractions were filtered through 0.22 μm PTFE (Polytetrafluoroethylene) filter for high-performance liquid chromatography coupled to diode array and mass spectrometry detector (HPLC-DAD-MS/MS) analysis. Additionally, the total content in phenolics, tannins and flavonoids were assessed by spectrophotometric assays, and the antioxidant capacity of each fraction was measured by 2.2’-diphenyl-1-picrylhydrazyl (DPPH·) radical-scavenging assay.

All the different fractions were dissolved in 100% ethanol to obtain stock solutions for cell experiments with the following concentrations: HF1 12.5 mg/mL, HF2 25 mg/mL, HF3 25 mg/mL and HF4 25 mg/mL. Extract doses used for cell treatments were considered on the basis of their cytotoxicity. Based on the total phenolic content of fractions, cells treated with 10 µg/mL, 25 µg/mL or 50 µg/mL HF (depending on the set of experiments) were considered to be exposed to 0.5μg GAE/mL^−2^ µg GAE phenolic fraction range /mL cell culture/ 106 cells.

### 2.4. HPLC-DAD Analysis

Chromatographic analyses of CBS fractions were performed with a HPLC-PDA Thermo-Finnigan Spectra System (Thermo-Finnigan, Waltham, USA) equipped with P2000 binary gradient pump, SCM 1000 degasser, AS 3000 automatic injector and Finnigan Surveyor PDA Plus detector.

The bioactive compounds were separated by reverse phase Kinetex Phenyl-Hexyl C18 column (150 × 4.6 mm internal diameter and 5 μm particle size) (Phenomenex, Castel Maggiore, Italy) thermostated at 35 °C. The mobile phase consisted of 0.1% formic acid (solvent A) and 100% methanol (solvent B). A gradient elution method was applied as follows: 0–2 min, 90% A and 10% B; 2–18 min, linear gradient from 10 to 50% B; 18–40 min, linear gradient from 50 to 80% B; 40–42 min, linear gradient up to 90% B; and 42–45 min 90% A and 10% B for column re-equilibration. The mobile phase flow rate was 1.0 mL/min and the sample injection volume was 10 μL. ChromQuest software (version 5.0) was used for instrument control, as well as data collection and processing. Data were acquired at 272 nm for theobromine and caffeine, 278 nm for epicatechin, catechin, type-B and type-A procyanidins, 293 nm for protocatechuic acid, and 350 nm for quercetin-3-*O*-glycosides. The quantification was assessed by the external standard method using six-point regression curves constructed at the wavelength of the maximum of absorbance of each molecule (*R*^2^ = 0.999).

### 2.5. Total Phenolics, Total Tannins and Total Flavonoids Contents

The total phenolic (TPC), flavonoid (TFC) and tannin (TTC) CBS fraction contents were determined according to the assays described by Barbosa-Pereira et al. [15]. The corresponding absorbance was recorded by using a BioTek Synergy HT spectophotometric multi-detection 96-well microplate reader (BiTek Instruments, Milan, Italy). All the determinations were performed in triplicate. The concentration of total phenolic compounds was determined using a standard curve of gallic acid (20–100 mg/L, *R*^2^ = 0.9981) and the results were expressed as mg of gallic acid equivalents (GAE)/L of fraction. As for TFC and TTC, the results were expressed as mg of catechin equivalents (CE)/L of fraction calculated on the basis of the standard curve of catechin (5–500 mg/L, *R*^2^ = 0.9980).

### 2.6. Antioxidant Capacity

CBS fraction radical scavenging activity (RSA) was evaluated by DPPH• radical scavenging assay as described by Barbosa-Pereira et al. [15]. The analyses were performed in triplicate using 96-well microplates, and RSA was evaluated by recording absorbance at 517 nm (BioTek Synergy HT spectophotometric multi-detection microplate reader, BioTek Instruments, Milan, Italy). The inhibition percentage (IP) of the radical DPPH was calculated using the following equation: IP (%) = [(A_0_ − A_30_)/A_0_] × 100, where A_0_ is the absorbance at time 0, and A_30_ is the absorbance after 30 min. The antioxidant capacity of the extracts was calculated as the equivalent EC_50_ concentration, which indicates the specific antioxidant activity of each fraction or extract expressed as mg equivalents of TPC/L fraction. EC_50_ represents the antioxidant concentration required to inhibit 50% of the initial DPPH radical, thus enabling the antioxidant activity comparison of all the fractions under the same experimental conditions.

### 2.7. RNA Extraction, cDNA Preparation and Real-Time Polymerase Chain Reaction (RT-PCR)

Total mRNA was extracted from differentiated CaCo-2 cells treated with Oxy-mix ± CBS fractions by using TRIzol. The concentration of extracted mRNA was determined by measuring the absorbance at 260 nm; 260/280 nm absorbance ratio was used to assess mRNA purity.

cDNA was synthesized by reverse transcription from 2 μg mRNA using a commercial kit and random primers (High-Capacity cDNA reverse transcription kit from Applied Biosystems, Monza, Italy), following the manufacturer’s instructions. Singleplex RT-PCR was performed on 20 ng cDNA using TaqMan gene expression assay kits prepared for human TLR2, TLR4 and β-actin. Amplified cDNAs were analyzed by using a 7500 Fast real-time PCR system (Applied Biosystems, Thermofisher, Monza, Italy). The oligonucleotide sequences are not revealed by the manufacturer because of proprietary interests. The cycling parameters were as follows: 40 cycles of 3 s each at 95 °C (melting) and 30 s at 60 °C (annealing/extension). The fractional cycle number at which fluorescence passes the threshold curve was determined for each considered gene. The results were normalized to the expression of β-actin, as housekeeping gene. Target gene expression was quantified as proposed by Livak and Schmittgen [26].

### 2.8. Cytokine Evaluation by ELISA

Collected culture medium from each cell treatment was used for the detection of IL-8, TNF-α, and IFN-β protein levels by using commercial ELISA kits (see Section 2.1. Chemicals) following the manufacturer’s instructions. Sample absorbance values were detected in a 96-multiwell plate reader (Model 680 Microplate Reader, Bio-Rad, Milan, Italy) using a dual-wavelength recording mode at 450 nm and 655 nm (the latter considered as reference). Data were analyzed by using SlideWrite Plus software (Advanced Graphics Software, Texas, USA). The analyses of the three different cytokines were performed in triplicate, normalized to total cell lysate protein concentration, and expressed as pg/mg of proteins/mL cell medium incubation. Medium protein concentration was evaluated by Bio-Rad protein assay dye reagent [27].

### 2.9. Statistical Analyses

Statistical differences among the different groups were evaluated using Student’s *t*-test and one-way ANOVA test. ANOVA associated Bonferroni’s multiple comparison post-test was performed to evaluate differences among the obtained molecular biology analysis values. Bonferroni correction is used to limit the possibility of getting a statistically significant result when testing multiple hypotheses. Therefore, this test was chosen as the most appropriate method in our biological models. On the other hand, ANOVA associated Duncan’s test was considered as the most suitable multiple range test to analyze differences among the values obtained by CBS extract chromatographic analyses. Data were processed with GraphPad Prism 6 software (San Diego, CA, USA) and results were expressed as mean ± Standard Deviation (SD).

## 3. Results

### 3.1. Oxysterols Induce TLR2 and TLR4 Gene Expression

Differentiated CaCo-2 cells were treated with 60 µM Oxy-mix in order to provide evidence of the potential ability of oxysterol to increase TLR2 and/or TLR4 expression.

**Figure 1:**
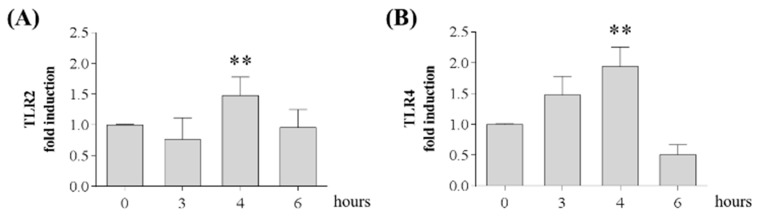
Oxy-mix induces TLR2 and TLR4 gene expression. TLR2 (panel **A**) and TLR4 (panel **B**) gene expression were evaluated through real-time RT-PCR in differentiated CaCo-2 cells treated with 60 µM Oxy-mix for 0, 3, 4 or 6 h. The significant maximum expression of both TLRs was reached after 4 h Oxy-mix cell treatment. Data are reported as means ± SD of three experiments performed in triplicate. One-way analysis of variance (ANOVA) associated with Bonferroni’s multiple comparison post-test was adopted: significantly different vs. 0 h: ** *p* < 0.01.

A time-dependent induction of TLR mRNA was detected by incubating cells with the Oxy-mix for 3, 4 and 6 h. As shown in Figure 1, TLR4 appeared to be activated earlier than TLR2 as it was already activated after 3 h (Figure 1B); however, the significant induction of both TLRs compared to control (Time 0) was only obtained after 4 h of cell incubation. TLR up-regulated expression by Oxy-mix was further confirmed by the detected receptors’ protein synthesis (Figure S2).

### 3.2. Oxysterol Pro-Inflammatory Effects Require TLR2/TLR4 Activation Involvement

**Figure 2:**
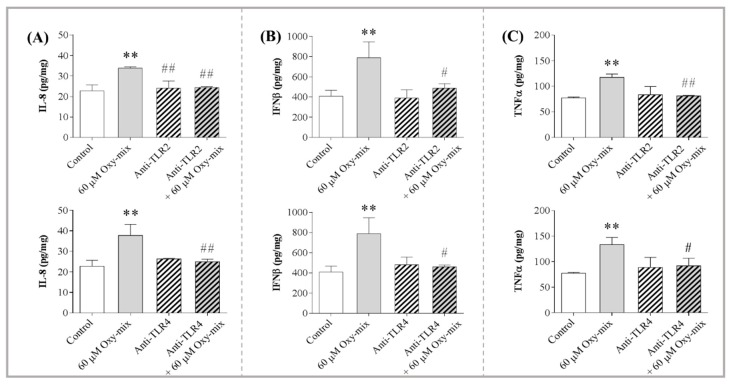
TLR2 and TLR4 are involved in Oxy-mix-dependent cytokine production. Differentiated CaCo-2 cells were pre-treated (1 h) with 0.2 μg/mL anti-TLR2 or anti-TLR4 antibodies, and then treated with 60 µM Oxy-mix for 24 h. IL-6 (panel **A**), IFNβ (panel **B**) and TNFα (panel **C**) levels were quantified by ELISA test in cell culture medium. Ab pre-treatment was able to fully prevent the Oxy-mix-dependent cytokine protein level increase in the cell medium. Values are shown as pg interleukin released/mg protein present in 1 mL cell incubation medium and are referred as means ± SD of three experiments performed in triplicate. One-way analysis of variance (ANOVA) associated with Bonferroni’s multiple comparison post-test was adopted: significantly different vs. controls (untreated cells): ** *p* < 0.01; significantly different vs. Oxy-mix: # *p* < 0.05 and ## *p* < 0.01.

IL-8, IFNβ and TNFα cell release in the incubation medium was considered as a parameter value of the oxysterol mixture’s ability to induce inflammation. The relationship between TLR expression and inflammation is well known. The involvement of TLR2 and 4 in the Oxy-mix dependent cytokine induction was assessed by using specific TLR2 or TLR4 antibodies. Differentiated CaCo-2 cells were treated with either 0.2 µg/mL anti-TLR2 or anti-TLR4 antibodies and IL-8; IFNβ and TNFα cell release was detected after 24-hour cell incubation with 60 µM Oxy-mix. The observed oxysterol-dependent increase of all the cytokines was lowered in the presence of specific antibodies against TLR2 or TLR4, suggesting the involvement of these receptors in mediating intestinal inflammation triggered by the Oxy-mix (Figure 2).

### 3.3. Differently Extracted CBS Fractions Show Different Antioxidant Activities Depending on Their Polyphenol Recovery

**Figure 3:**
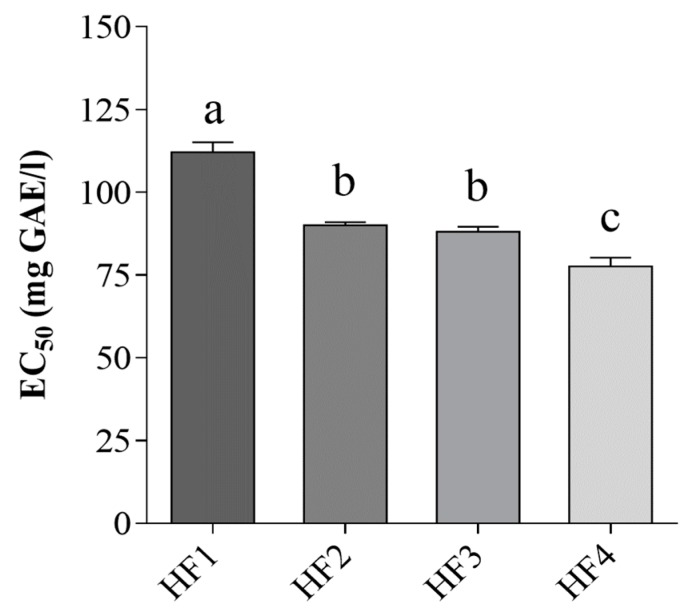
Antioxidant capacity of different CBS extract fractions from Honduras. Antioxidant capacity was analyzed by performing DPPH· radical scavenging assay. The highest antioxidant activity was recorded in HF4 analysis. The results are reported as EC_50_ equivalent concentration and expressed as mg/L (mg equivalents of TPC/L of fraction). Data are reported as mean values (*n* = 3) ± SD. One-way analysis of variance (ANOVA) with Duncan’s test was used as a multiple range test to highlight the significant differences among all the treatments. ANOVA was considered significant for *p* < 0.001. Means with different lowercase letters within the same column are significantly different (Duncan’s test, *p* < 0.05): ^a^ different from HF2, HF3, HF4; ^b^ different from HF1, and HF4; ^c^ different from HF1, HF2, HF3.

The fractionation method of CBS extract by solid-phase extraction allowed the recovery of four fractions (called HF1, HF2, HF3, HF4) with different polyphenolic contents that were analyzed by HPLC-DAD (see Materials and Methods). The chromatographic results revealed that the elution of CBS extract with two different solvents (100% methanol or acetone-water (70:30 *v*/*v*)) produced two fractions, HF3 and HF4, which showed the highest amount of phenolic compounds. Different constituents of the four fractions are reported in Table 1. HF3, indeed, is rich in (−)-epicatechin and total flavonoids, while HF4 was found to be characterized by high amounts of tannin such as procyanidin B-type (PCB) and Procyanidin A-type Pentoside (PCA Pentoside). However, since HF4 was the fraction richest in tannins, further analyses involving MS spectrometry detectors such as MALDI-TOF need to be done to identify procyanidins with a high degree of polymerization that may be present in CBS. Notably, HF1 showed the highest theobromine concentration (Table 2). However, with regard to antioxidant activity, only HF1 was unable to reduce DPPH compared to the other three fractions (Figure 3). The main compounds present in high amounts in the fractions were identified and quantified. However, other molecules could be present in the extracts that were not possible to identify, and which could also contribute to the bioactivity displayed by fractions [28].

### 3.4. HF3 and HF4 (but not HF1 and HF2) Prevent Oxysterol-Mediated Inflammation in Terms of IL-8 Cell Release

Differentiated CaCo-2 cells were pre-treated (1 h) with increasing concentrations of HFs (10 µg/mL, 25 µg/mL and 50 µg/mL) and then incubated with 60 µM Oxy-mix for 24 h. Lactate Dehydrogenase (LDH) cell release was evaluated in order to examine possible cytotoxicity of the different compound concentrations. The highest concentration of all tested HFs markedly increased LDH percentage cell release compared to the lower concentrations (Supplementary Materials Table S1).

Based on this evidence, we carried out experiments aimed to evaluate oxysterol and HF ability to modulate IL-8 levels in to cell medium by using HF concentrations of 10 µg/mL and 25 µg/mL.

**Figure 4:**
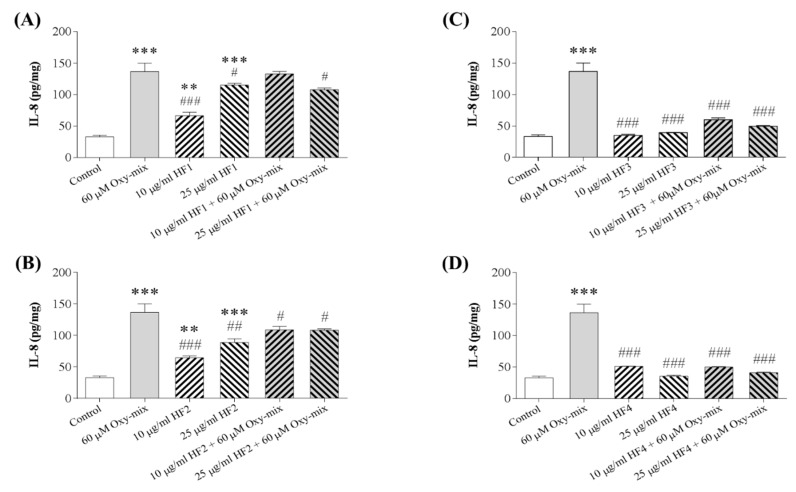
HF3 and HF4 prevent IL-8 cell release induced by oxysterol treatment. Differentiated CaCo-2 cells were pre-treated with 10 or 25 µg/mL HF1 (**A**), HF2 (**B**), HF3 (**C**) or HF4 (**D**) for 1 h, then incubated with 60 µM Oxy-mix for 24 h. IL-8 cell release in the incubation medium was considered as a marker of inflammation. Cytokine was evaluated by ELISA test and was expressed as pg/mg proteins/mL cell incubation medium. HF3 and HF4 were able to fully prevent IL-8 production with both 10 and 25 µg/mL concentrations. IL8 was already increased in the medium from the cells treated with HF1 and HF2 and not with Oxy-mix, while cytokine basal levels were not affected by HF3 or HF4. Data are reported as means ± SD of three experiments performed in triplicate. One-way analysis of variance (ANOVA) associated with Bonferroni’s multiple comparison post-test was adopted: significantly different vs. controls: ** *p* < 0.01 and *** *p* < 0.001; significantly different vs. Oxy-mix: # *p* < 0.05, ## *p* < 0.01 and ### *p* < 0.001.

The incubation with 60 µM Oxy-mix for 24 h induced a strong interleukin increase. Among the four HFs, only HF3 and HF4 were able to fully prevent IL-8 induction with both 10 and 25 µg/mL concentrations (Figure 4).

Notably, data from cell pre-treatment with different fractions in the absence of Oxy-mix showed that HF3 and HF4 did not influence IL-8 medium protein basal levels (Figure 4C,D). On the contrary, HF1 and HF2 were able to significantly increase IL-8 production even without Oxy-mix incubation (Figure 4A,B). Probably, the inflammatory effects of HF1 and HF2 fractions should be ascribed to their theobromine and caffeine content, neither of which were detected in HF3 and HF4, as previously described in the above Table 1.

### 3.5. HF3 and HF4 Prevent TLR2 and TLR4 Oxy-Mix Induction

A set of experiments was performed in order to verify if HF3 and/or HF4 could prevent oxysterol-mediated inflammation through the action on TLRs.

**Figure 5:**
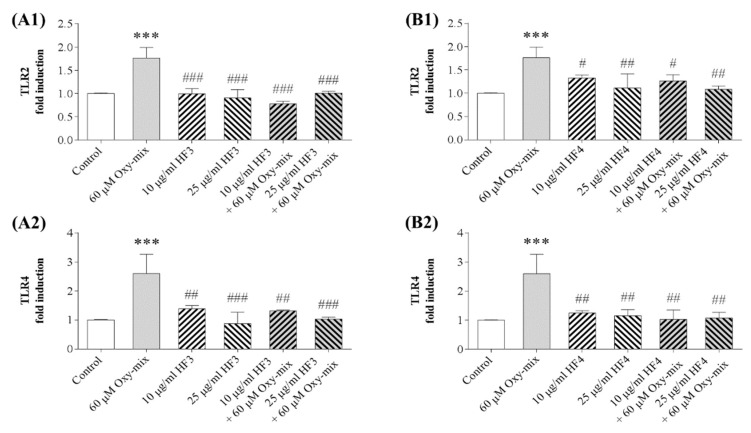
HF3 and HF4 prevent TLR2 and TLR4 induction by dietary oxysterols in differentiated CaCo-2 cells. HF3 (Panel A): TLR2 (**A1**), TLR4 (**A2**); HF4 (Panel B): TLR2 (**B1**), TLR4 (**B2**). Cells were pre-treated or not with 10 or 25 µg/mL of HF3 or HF4 for 1 h. Cells were then treated with 60 µM Oxy-mix for 24 h. TLR2 and TLR4 expression was evaluated by using RT-PCR. CBS extract pre-treatment significantly prevented the gene expression induction of both TLR2 and TLR4. Data are reported as means ± SD of three experiments performed in triplicate. One-way analysis of variance (ANOVA) associated with Bonferroni’s multiple comparison post-test was adopted: significantly different vs. controls: *** *p* < 0.001; significantly different vs. Oxy-mix: # *p* < 0.05, ## *p* < 0.01 and ### *p* < 0.001.

Differentiated CaCo-2 cells incubated with 60 µM Oxy-mix, but pre-treated with 10 or 25 µg/mL HF3 (Figure 5A) or HF4 (Figure 5B), showed a significantly decreased expression of both TLR2 (Figure 5A1,B1) and TLR4 (Figure 5A2,B2) compared to the Oxy-mix sample, suggesting their distinct role in also counteracting the effects of oxysterol.

## 4. Discussion

Dietary-absorbed oxysterols, absorbed as products of cholesterol auto-oxidation have been suggested to potentially interfere with epithelial barrier function by promoting and sustaining inflammation [10]. We have recently demonstrated the ability of dietary oxysterols to affect CaCo-2 cell monolayer permeability by using the same combination and concentration of oxysterols employed in the present study [12]. The observed alteration of tight junction cell levels and distribution was associated with the ability of pro-inflammatory oxysterols to activate matrix metalloproteinases, which are likely responsible for intestinal layer derangement [12].

A wide range of literature underlines TLR dysfunction and dysregulation in intestinal epithelial cells as crucial for IBD development and progression. Increased expression of certain TLRs in IBD may result from their hyper-activation by microbiota, or may be a secondary event to inflammatory reactions and intestinal layer damage [29].

Therefore, it would be plausible to hypothesize that these receptors could be involved in the intestinal mucosa inflammatory damage induced by a diet rich in oxysterols. In our study, RT-PCR analyses showed increased TLR2 and TLR4 expression after 4 h differentiated CaCo-2 cell incubation with 60 µM Oxy-mix, a dose corresponding to high cholesterol intake, which has consistently been demonstrated to be remarkably pro-inflammatory [11].

The ability of specific oxysterols to bind directly to cell surface receptors, thus activating their related cell signaling pathways, has been specifically demonstrated for enzymatically produced oxysterols such as 27-hydroxycholesterol or 25-hydroxycholesterol. These compounds are involved in lipid metabolism and reverse cholesterol transport, being very good ligands for Liver X Receptors [30,31]. 27-hydroxycholesterol was also considered for its tumor promoting action by binding to and activating estrogen receptor (ER) pathways in breast cancer cells [32]. Notably, it was able to activate inflammation by binding to TLR4 in promonocytic U937 cells. Interestingly, in this study 27-hydroxycholesterol was found to enhance cell release of IL-8, IL-1β, and TNF-α and to upregulate matrix metalloproteinase-9 (MMP-9) via the TLR4/NF-κB-dependent pathway, a phenomenon that has been associated to atherosclerotic lesion worsening during the process of atherosclerosis [33].

There is some evidence regarding the capacity of exogenously produced oxysterols in activating TLR-dependent signals. Among different oxysterols of dietary origin, 7-ketocholesterol is the most represented oxysterol in our experimental mixture. 7-ketocholesterol activates numerous inflammatory pathways in different cell types. Aye and colleagues reported the ability of this sterol to exert pro-inflammatory action via TLR4 activation in placental trophoblasts [34]. A study by Huang and collaborators showed that maximum 7K-induced inflammation occurs via TLR4 pathway both in vitro and in vivo in immortalized human retinal pigment epithelium (RPE) cells and in Male Brown Norway rat eyes, respectively [35]. On the other hand, IL-8 induction by 7K, 7β-HC or 25-hydroxycholesterol appeared to be independent of TLR activation in THP-1 macrophages, including TLR2 and 4. However, this was demonstrated by using other cytotypes: TLR transfection of human embryonic kidney (HEK)-293 cells, which constitutively lack TLR expression, did not confer IL-8 increased sensitivity to these three oxysterols. Additionally, only 25-hydroxycholesterol and not 7-KC or 7β-HC, increased Interleukin promoter transcriptional activity by using full-length IL-8 promoter in the same experimental model in the absence of TLR expression [36]. A more recent study showed that the strong induction of IL-8 expression by 7α-HC in THP-1 cells was TLR2 and TLR4 independent [37].

In the present study, our findings on differentiated intestinal CaCo-2 cells underline that the differential response to oxysterols depends on the cell types used as the experimental model. Notably, the intestinal layer is an active participant in the control of tolerance and immunity in response to luminal antigens. The active involvement of TLR2 and 4 in the induction of inflammatory cytokines triggered by a mixture of dietary oxysterols in intestinal cells was demonstrated by the use of specific TLR2 or TLR4 antibodies. Cell pre-treatment with these antibodies significantly prevented 60 µM Oxy-mix-dependent inflammation in terms of decreased cell release of IL-8, TNF-α and IFN-β in culture medium.

Data suggest oxysterols can act straight on these receptors, although there is no clear evidence of their direct function as TLR ligands. It is noteworthy that oxysterols can enter lipid rafts by displacing cholesterol, thus altering their composition and function and subsequent cell surface receptor activity. For instance, they might promote a non-canonical receptor pathway activation by interfering with lipid microdomains necessary for recruitment and clustering of TLR activation complexes [38,39].

The anti-inflammatory activities of polyphenol extracts from green tea, cocoa and red wine polyphenols in colon cells exposed to lipopolysaccharide have been demonstrated [40]. As reported in our previous studies, several phenolic compounds specifically counteract the inflammatory action of oxysterols in colon cells. Regarding this, epigallocatechin-3-gallate, (−)-epicatechin, and caffeic acid were able to decrease Oxy-mix-induced inflammation and permeability derangement in differentiated CaCo-2 cells [10,12,41]. Cocoa is known to be rich in (−)-epicatechin [42,43], and its consumption has exponentially increased in the last decade. Scientific literature has reported that this compound is able to decrease inflammation in several human diseases, such as cardiovascular, Parkinson’s, Alzheimer’s and intestinal diseases [44,45,46]. For instance, cocoa was shown to counteract inflammation in an in vivo model of obesity with high fat-fed mice [47]. Cocoa procyanidins have been found to possess distinct anti-inflammatory effects on colon cell inflammation depending on their degrees of polymerization [28].

Cocoa bean utilization in food industries produces thousands of tons of shells every year, representing one of the most relevant worldwide waste issues. This by-product first gained attention for being rich in phenolic compounds [48], which could be recycled as food supplements. In order to obtain the maximum concentration of flavonoids from cocoa bean shells, purification methods are crucially important.

Therefore, we provided four different extraction methods of CBS produced from Honduras cocoa beans, which showed four fractions with different phenolic compositions. In particular, (−)-epicatechin and tannins were higher in HF3 and HF4 than HF1 and HF2. This suggests that HF3 and HF4 are potentially more efficient than the other extracts in their anti-inflammatory action for their specific polyphenol content. In addition, HF1 did not show any significant antioxidant capacity as was detected for HF2 and HF3, and strongly induced by HF4. HF1’s lack of antioxidant capacity could be due to its high theobromine concentration. Notably, this fraction also showed increased LDH release compared to the other fractions.

The data reported here showed the strong activity of HF3 and HF4 in quenching both IL-8 hyper-production and TLR over-expression in cells treated with Oxy-mix, highlighting the important presence of high amounts of (−)-epicatechin and tannins in these two extracts.

The mechanism of action of these polyphenols against oxysterol-dependent induction of TLR-mediated inflammatory signals might be due to several factors. Based on the evidence that Oxy-mix has a strong pro-oxidant effect in differentiated CaCo-2 cells [49], HF3 and HF4 anti-inflammatory action could be partly due to their antioxidant capacity, thus negatively modulating TLR expression. On the other hand, these compounds could limit oxysterol accessibility to the membrane by interacting with lipid rafts, where specific membrane proteins necessary for receptor activation—including TLRs and NADPH oxidase—are located [50,51]. Notably, a direct interaction between TLR4 and NADPH oxidase in LPS-induced production of oxidant species has been demonstrated [52].

## 5. Conclusions

The reported findings in our experimental model of intestinal cell monolayer strikingly indicate that a combination of oxysterols similar to that found in heat processed cholesterol-rich food can cause intestinal inflammation through increased expression and activation of TLR2 and TLR4 receptors. Furthermore, the preventive inhibition of IL-8, as well as TLR2 and TLR4 by HF3 and HF4 CBS fractions demonstrates that these extracts could interfere with oxysterol-mediated inflammation thanks to their high content of polyphenols.

We would like to emphasize that a detailed characterization of bioactive compounds of cocoa residues may pave the way to a zero-waste policy for cocoa industry. CBS could become a desirable raw material for a wide range of applications, including the health field as its antioxidant and anti-inflammatory properties show great potential as a food ingredient.

## Figures and Tables

**Table 1:** Main composition of HFs.

Compounds	HF1 (µg/mL)	HF2 (µg/mL)	HF3 (µg/mL)	HF4 (µg/mL)
**Methylxanthines**				
Theobromine	8045.3 ± 43.3	198.2 ± 1.6	N.Q.	N.Q.
Caffeine	1390.0 ± 17.7	10.0 ± 0.3	N.Q.	N.Q.
**Polyphenols**				
Protocatechuic acid	N.Q.	92.0 ± 2.6	85.8 ± 2.1	N.Q.
PCB2	N.Q.	186.4 ± 2.6	N.Q.	N.Q.
Catechin	N.Q.	N.Q.	181.1 ± 2.8	N.Q.
Epicatechin	N.Q.	N.Q.	1339.7 ± 45.8	N.Q.
PCA pentoside	N.Q.	N.Q.	100.8 ± 1.9	83.0 ± 1.5
PCB	N.Q.	N.Q.	N.Q.	743.6 ± 3.20
Quercetin-3-glucoside	N.Q.	N.Q.	53.4 ± 2.0	N.Q.
Quercetin-3-arabinside	N.Q.	N.Q.	95.8 ± 1.3	47.0 ± 1.2

The analysis of HF1, HF2, HF3 and HF4 was performed by HPLC-DAD (see Materials and Methods, Section 2.4). HF3 and HF4 were the richest in polyphenol content. Data are expressed as mean values (*n* = 3) ± SD. N.Q.: Not quantified by HPLC-DAD.

**Table 2:** Analysis of polyphenols present in Honduras CBS fractions (HFs).

Fraction	TPC (mg GAE/L)	TFC (mg CE/L)	TTC (mg CE/L)
**HF1**	546.7 ± 27.3 ^a^	90.6 ± 0.5 ^a^	85.0 ± 5.6 ^a^
**HF2**	416.2 ± 9.7 ^b^	280.3 ± 8.5 ^d^	120.0 ± 0.8 ^d^
**HF3**	425.7 ± 7.6 ^b^	296.9 ± 8.1 ^e^	243.5 ± 1.9 ^e^
**HF4**	372.5 ± 29.4 ^c^	222.0 ± 9.3 ^c^	359.7 ± 6.0 ^c^
*Significance*	***	***	***

Total phenolic content (TPC), total flavonoid content (TFC) and total tannin content (TTC) concentrations were evaluated as described by Barbosa-Pereira and colleagues [15]. Concentration of TPC is expressed as mg of GAE/l of fraction, while TFC and TPC are reported as mg of catechin equivalents (CE)/l fraction. Data are expressed as mean values (*n* = 3) ± SD. One-way analysis of variance (ANOVA) with Duncan’s test was used as a multiple range test to highlight the significant differences among all of the treatments. Means with different lowercase letters within the same column are significantly different (Duncan’s test, *p* < 0.05): ^a^ different from HF2, HF3, HF4; ^b^ different from HF1, and HF4; ^c^ different from HF1, HF2, HF3; ^d^ different from HF1, HF3, HF4; ^e^ different from HF1, HF2, HF4: *p* < 0.05. ANOVA significance: *** *p* < 0.001.

## Supplementary Materials

The following are available online at https://www.mdpi.com/2076-3921/8/6/151/s1, Figure S1. Main oxysterols derived from cholesterol autoxidation present in the diet. Figure S2. Oxy-mix induces TLR2 and TLR4 protein levels. Table S1. Cytotoxic effects of Honduras CBS fractions with or w/o oxysterol cell treatments.

## Abbreviations

**α-epox**	5α,6α-epoxycholesterol
**β-epox**	5β,6β-epoxycholesterol
**7α-HC**	7α-hydroxycholesterol
**7β-HC**	7β-hydroxycholesterol
**7K**	7-ketocholesterol
**CBS**	Cocoa bean shells
**cDNA**	Complementary DNA
**CE**	Catechin equivalents
**DAMPs**	Damage-associated molecular patterns
**DMEM**	Dulbecco's modified Eagle's medium
**DPPH•**	2,2’-diphenyl-1-picrylhydrazyl
**ELISA**	Enzyme-Linked Immunosorbent Assay
**ER**	Estrogen receptor
**FBS**	Fetal bovine serum
**GAE**	Gallic acid equivalents
**HEK-293**	Human embryonic kidney cells
**HF1**	Honduras-Fraction 1
**HF2**	Honduras-Fraction 2
**HF3**	Honduras-Fraction 3
**HF4**	Honduras-Fraction 4
**HPLC-DAD-MS/MS**	High-performance liquid chromatography coupled to diode array and mass spectrometry detectors
**IBDs**	Inflammatory bowel diseases
**IFN-β**	Interferon-β
**IL**	Interleukins
**IP**	Inhibition percentage
**LDH**	Lactate Dehydrogenase
**MMP**	Matrix metalloproteinase
**Oxy-mix**	Oxysterol mixture
**PAMPs**	Pathogen-associated molecular patterns
**PCA**	Procyanidin A-type
**PCB**	Procyanidin B-type
**PCB2**	Procyanidin B2
**PCR**	Polymerase chain reaction
**PTFE**	Polytetrafluoroethylene
**RPE**	Retinal pigment epithelium
**RSA**	Radical scavenging activity
**RT-PCR**	Real-time polymerase chain reaction
**SPE**	Solid-phase extraction
**TFC**	Total flavonoid content
**TLRs**	Toll-like receptors
**TNF-α**	Tumor necrosis factor-α
**TPC**	Total phenolic content
**TTC**	Total tannin content
