# Supplementary material for: A Dietary Mixture of Oxysterols Induces In Vitro Intestinal Inflammation through TLR2/4 Activation: The Protective Effect of Cocoa Bean Shells
Source: Antioxidants (Basel). 2019 May 31;8(6):151. doi: 10.3390/antiox8060151 (PMC6617147; doi:10.3390/antiox8060151)
Supplement: Supplementary file 1 [file antioxidants-08-00151-s001.pdf]

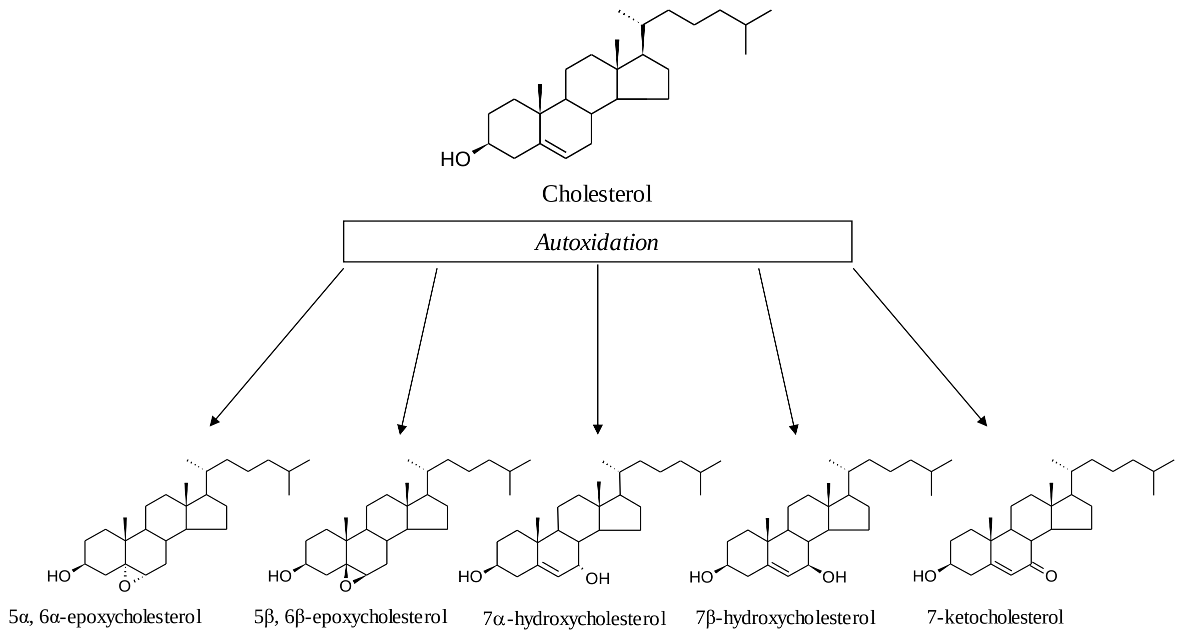

Figure S1. Main oxysterols derived from cholesterol autoxidation present in the diet.

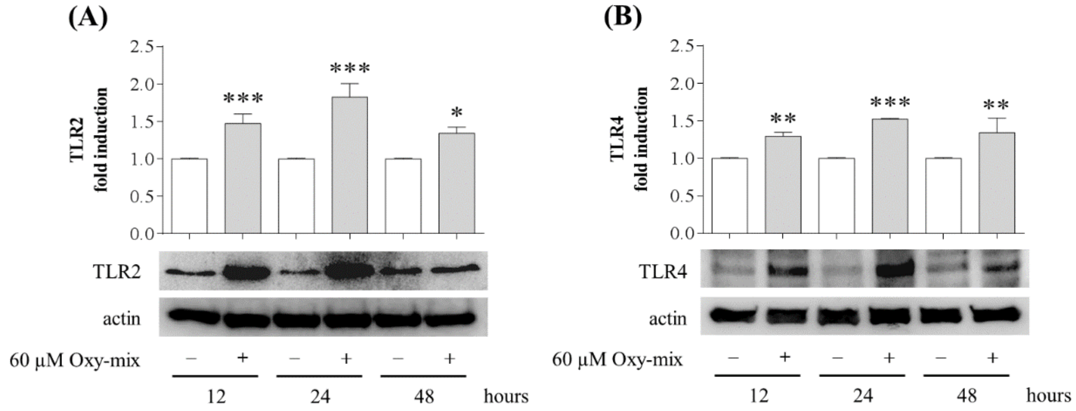

Figure S2. Oxy-mix induces TLR2 and TLR4 protein levels. TLR2 (panel **A**) and TLR4 (panel **B**) proteins were evaluated through Western Blotting in differentiated CaCo-2 cells treated with 60 µM Oxy-mix for 12, 24 or 48 hours. Data are reported as means ± SD of three experiments. One-way analysis of variance (ANOVA) associated with Bonferroni's multiple comparison post-test was adopted: significantly different vs. control (untreated cells): *$p < 0.05$; **$p < 0.01$; ***$p < 0.001$.

Table S1. Cytotoxic effects of Honduras CBS fractions with or w/o oxysterol cell treatments.

**LDH Cell Release (%)**		
	**---**	**60 µM Oxy-Mix**
---	7 ± 2.3	9.2 ± 4.3
**10 µg/mL HF1**	6.9 ± 1.9	9 ± 1.6
**25 µg/mL HF1**	9.3 ± 1.6	20 ± 3.9 $^{\#}$
**50 µg/mL HF1**	28.9 ± 9.5 ***	25 ± 8.5 $^{***;\#\#}$
**10 µg/mL HF2**	11.2 ± 2.8	18.8 ± 2.8
**25 µg/mL HF2**	16.2 ± 8.4	19.1 ± 6.1
**50 µg/mL HF2**	15.3 ± 11.1	16.8 ± 2.5

**10 μg/mL HF3**	6.1 ± 1.3	6.5 ± 1.6
**25 μg/mL HF3**	7 ± 1.7	7.5 ± 0.6
**50 μg/mL HF3**	17.1 ± 2.1	10.3 ± 6.1
**10 μg/mL HF4**	12.6 ± 3.6	11.3 ± 2.5
**25 μg/mL HF4**	16.6 ± 3.7	16.7 ± 2.1
**50 μg/mL HF4**	18.1 ± 2.4	17.8 ± 1.4

Cytotoxicity was evaluated as percentage of lactate dehydrogenase (LDH) total release into cell medium. Differentiated CaCo-2 cells were pretreated with 10, 25 or 50 μg/ml Honduras fractions (HF) for 1 hour and treated with 60 μM Oxy-mix for 24 hours. Data are reported as means ± SD of three experiments performed in quadruplicate. One-way analysis of variance (ANOVA) associated with Bonferroni's multiple comparison post-test was adopted: significantly different vs. control (untreated cells): ****p* < 0.001; significantly different vs. Oxy-mix: #*p* < 0.05, ##*p* < 0.01.
